# Association of eNOS (G894T, rs1799983) and KCNJ11 (E23K, rs5219) gene polymorphism with coronary artery disease in North Indian population

**DOI:** 10.4314/ahs.v21i3.25

**Published:** 2021-09

**Authors:** Syed Tasleem Raza, Sachendra P Singh, Saliha Rizvi, Alina Zaidi, Sanchita Srivastava, Arif Hussain, Farzana Mahdi

**Affiliations:** 1 Department of Biochemistry, Era's Lucknow Medical College and Hospital, Lucknow, India; 2 Department of Molecular biology, Manipal Academy of Higher Education

**Keywords:** Coronary Artery Disease (CAD), endothelial nitric oxide synthase (eNOS), potassium voltage-gated channel subfamily J member 11 (KCNJ11), gene polymorphism

## Abstract

**Background:**

Endothelial nitric oxide synthase (eNOS) and potassium voltage-gated channel subfamily J member 11 (KCNJ11) could be the candidate genes for coronary artery disease (CAD). This study investigated the relationship of the eNOS (rs1799983) and KCNJ11 (rs5219) polymorphisms with the presence and severity of CAD in the North Indian population.

**Methods:**

This study included 300 subjects, 150 CAD cases and 150 healthy controls. Single nucleotide polymorphism was evaluated by Polymerase chain reaction and Restriction fragment length polymorphism (PCR-RFLP). Analysis was performed by SPSS (version 21.0).

**Results:**

We observed that KK genotype of KCNJ11E23K (rs5219) polymorphism (P=0.0001) genotypes and K allele (P=0.0001) was found to be a positive risk factor and strongly associated with CAD. In the case of eNOSG894T (rs1799983) there was no association found with CAD.

**Conclusion:**

These results illustrate the probability of associations between SNPs and CAD although specific genetic polymorphisms affecting ion channel function and expression have still to be clarified by further investigations involving larger cohorts.

## Background

Coronary artery disease (CAD) is an ischemic heart disease which has been found to be the major cause of mortality in both developed and developing countries^1^. This atherosclerotic disease is inflammatory in nature^2^ with clinical manifestations which include both stable and unstable angina, myocardial infarction (MI), and sudden cardiomyocyte death^3^. According to genome-wide association studies (GWAS), numerous genetic mutations have also been found to be robustly allied with CAD^4^. Other worldwide studies conducted on the genetic variations in CAD risk among populations reported that the disease incidence is also attributable to demographic phenomenon^5^, ^6^. Additionally, lifestyle also plays a profound role in the progression of such cardiovascular events^7^. World Health Organization in the year 2009 documented through several reports that 17.3 million deaths prevailed due to cardiovascular diseases^2^. Moreover, among Indian population, CAD has been the chief cause of disability and death^8^ and has been the source of mortality leading to increase number of deaths from the year 1985 which was expected to be doubled by the year 2015^9^. However, therapeutic measures have significantly enhanced the prognosis of CAD patients over the past few decades^10^. Yet, the progression of the disease could be halted only in a few patients via treatments through aspirin, statins, and β-blockers^11^. By 1879, it was concluded by pathologists that CAD was the basis of myocardial infarction (MI)^12^.

The CAD occurs due to atherosclerosis or atherosclerotic occlusions of the coronary arteries^13^. Endothelial dysfunction of the arterial wall due to the accumulation of modified low-density lipoprotein (LDL) in the intima of the coronary vessels gives rise to atherosclerosis^14^. High concentration of LDL permeates the disrupted endothelium and undergoes oxidation^15^ which draws leukocytes which are further scavenged by macrophages, leading to the development of foamy cells. These foamy-texture cells undergo replication and form lesions which aid in early detection of atherosclerosis. This cascade triggers signals which attract smooth muscle cells (SMCs) to this site and provoke their proliferation and production of extracellular matrix (ECM), including collagen and proteoglycans which further initiates atherosclerotic fibrous plaque and encroaches on the lumen of the coronary vessel and small blood vessels subsequently calcifying the plaques thus forming final lesion that may be highly thrombogenic^16^. This is followed by obstruction of blood flow leading impaired myocardial oxygen demand and supply^17^. These events give rise to the symptoms related to CAD.

Nitric oxide synthase (NOS) is one of the chief candidate genes in CAD. It synthesizes NO in a catabolic reaction in presence of L-arginine^18^. The gene is located on chromosome 7q36. The three isoforms of NOS are neuronal isoforms (nNOS), inducible isoform (iNOS) and endothelial NOS (eNOS). The capability of a blood vessel to dilate is largely dependent upon the activity of eNOS hence the present article will focus on this isoform as one of the candidate gene. Endothelial NOS (eNOS), also known as nitric oxide synthase 3 (NOS3) or constitutive NOS (eNOS), is an enzyme in humans which is encoded by the NOS3 gene located in the 7q35-7q36 region of chromosome 7,19. eNOS also acts as potent regulator of blood pressure and blood flow^20^. Elevated levels of iron could lead to free hydroxyl radicals synthesis resulting in LDL oxidation^21^ which is considered as one of the prime factor in pathogenesis of atherosclerosis and cardiovascular diseases^22^ due to a lipid accumulation in macrophages and foam cells, thus conferring toxicity to cells.

Another gene that has been focused in this article is KCNJ11 gene as it also guides the formation of ATP-sensitive potassium (K-ATP) channel in cardiomyocytes. These ATP sensitive channel play important role in CAD^23^. Usually, the K-ATP channels in cardiac tissue are closed when the intracellular ATP concentration is high^24^, but get activated in cardiovascular pathological states which include ischemia, reperfusion, cell stress and apoptosis^25^,^26^ due to which the cellular membrane hyperpolarization occurs which promotes te synthesis of NO thus increasing the permeability of the vascular wall. This leads to the pathologic development of coronary atherosclerosis. Any mild mutations or polymorphisms modifying the KATP channel current or activity will be correlated with diseases. It was reported that the common polymorphism E23K of this gene is associated with higher susceptibility to CAD in many population.

The purpose of this study is to disclose the relationship between G894T (rs1799983) polymorphisms of the eNOS gene and KCNJ11 E23K (rs5219) with the presence of CAD in North Indian population. Single nucleotide polymorphisms (SNPs) could serve as prognostic factor as they influence disease incidence and can be employed in everyday clinical practice and decision making; hence, it is essential to identify those SNPs that have strongest predisposition. Location of SNPs play influential role in incidence of diseases. Any variation of the SNP could be directly connected to the disease if it is located within or close to the translated region. As a result, various research studies envisaged the identification of candidate and sensitive genes for CAD and their polymorphisms in relation to the risk of this disease.

## Methods

### Subject's enrollment

This case-control study was performed on 300 subjects including 150 CAD cases (who underwent coronary angiography) and 150 controls were recruited. Blood samples were collected in anti-coagulation EDTA vials from the Cardiology Unit (Department of Medicine), Era's Lucknow Medical College & Hospital of Lucknow, Uttar Pradesh. A written informed consent was obtained from all participants prior to sampling. All the baseline clinical prolific collection for each patient was done that included clinical variable such as age, sex, blood pressure, body mass index, height, weight, lipid profile etc. The diagnosis of CAD was defined as > 50% reduction of coronary artery diameter. Cases were classified according to the number of significant stenotic vessels as follows: angigraphically-normal vessel (n = 19), 1-vessel (SVD) (n = 44), 2-vessel (DVD) (n = 58) and 3- vessel (TVD) (n = 64). Control subjects (n=160) were defined as those individuals who had no personal or family history of cardiovascular disease or diabetes. Protocol and procedures were in accordance with the standards of the Institutional Ethical/ Review Committee.

### Genomic DNA isolation

Genomic DNA isolation from peripheral blood samples was done using the standard phenol-chloroform extraction method and was checked on 1% agarose gel stained with ethidium bromide. Quantification of the extracted DNA was done using Nanodrop (TM) 1000 UV/VIS Spectrophotometer. Finally the isolated DNA was stored at -20°C until further analysis was done.

### PCR Amplification

**Endothelial nitric oxide synthase (eNOS) (G894T, rs1799983) Gene Polymorphism**

**Forward Primer: 5′ CATGAGGCTCAGCCCCAGAAC 3′**

**Reverese Primer: 5′ AGTCAATCCCTTTGGTGCTCAC 3′**

Approximately 100 ng of genomic DNA was amplified in a total volume of 25 µL containing 2.5 µL of thermophilic DNA Polymerase Buffer (supplied with Taq polymerase; Promega), 3.0 mmol/L MgCl2, 200 µmol/L deoxynucleotide triphosphates, 10 pmol/L of each primer/span> and 1 U of Taq polymerase.

The PCR amplification conditions were 95 °C for 5 mins followed by 40 cycles of denaturation at 94 °C for 30 s, annealing at 66 °C for 30 s, extension at 72 °C for 30 s. The final extension step was carried out at 72 °C for 7 min. The 206-bp PCR product (10 µl) was digested with MboI restriction endonuclease (NEB, UK) overnight and the digested products were analyzed on 3% agarose gel and visualized by ultraviolet transillumination after ethidium bromide staining. The 206 bp amplicon containing a thymine at nucleotide position 894 (corresponding to an aspartic acid at amino acid position 298, TT genotype) was cleaved into two fragments of 119 bp and 87 bp in length by MboI digestion but not for a guanine in this position (206 bp, GG genotype).

Potassium voltage-gated channel subfamily J member 11(KCNJ11) E23K (rs5219) Gene Polymorphism

Forward Primer: 5′ GACTCTGCAGTGAGGCCCTA 3′

**Reverse Primer:** 5′ ACGTTGCAGTTGCCTTTCTT 3′

Approximately 100 ng of genomic DNA was amplified in a total volume of 25 µL containing 2.5 µL of Thermophilic DNA Polymerase Buffer (supplied with Taq polymerase; Promega), 3.0 mmol/L MgCl2, 200 µmol/L deoxynucleotide triphosphates, 10 pmol/L of each primer and 1 U of Taq polymerase.

The PCR amplification conditions were 95 °C for 5 mins followed by 40 cycles of denaturation at 94 °C for 30 s, annealing at 66 °C for 30 s, extension at 72 °C for 30 s. The final extension step was performed at 72 °C for 7 min. The 210-bp PCR product (10 µl) was digested with Ban II restriction endonuclease (NEB, UK) for 1 hr at 37°C and the digested products were analyzed on 3% agarose gel and visualized by ultraviolet transilluminatior after ethidium bromide staining.

### Statistical analysis

QUANTO (v.online) software for each single nucleotide polymorphism (SNP) was used to calculate the sample size. Minor allelic frequency (MAF) and distribution were used for evaluation. The Hardy-Weinberg equilibrium for each locus is evaluated by the Chi-square test (X2). The continuous variables for each group were expressed as mean ± SD and compared with the Student's t test after determining the normality of the Kolmogorov-Smirnov Z test. In both groups, the allele frequencies were compared by comparing the 2 x 2 matrix. P <0.05 was considered statistically significant. The 95% confidence interval (CI) ratio was determined in the logistic regression model to describe the strength of the two SNPs. All analyzes were performed by SPSS (version 21.0).

## Results

### Restriction fragment length polymorphism analysis for determination of genotype

For eNOS gene polymorphisms, the GT genotype shows two bands of base pairs 206 bp and 119 bp. GG genotype shows single band of 206 bp and TT genotype shows two bands of 119 bp and 87bp. In case of KCNJ11 gene polymorphisms, UD shows undigested band of 210bp, EK genotype shows three bands of 210 bp, 178 bp and 150 bp; EE genotype shows one band of 150 bp and KK genotype shows two bands of 178 bp and 150 bp.

### Demographic and clinical characteristics

Demographic, clinical and biochemical characteristics of the studied population were recorded from both cases and controls. The mean age of cases and controls were 52.68±5.440 and 52.00±5.188 respectively (P=0.269). Clinical parameters such as BMI (P=0.005), LDL(0) and total cholesterol (0) was significantly higher in cases as compared to controls whereas, HDL (p<0.001), Triglyceride (p=0.003)and VLDL(p=0.003) level was found to be significantly lower in cases as compared to controls (Table 1).

**Table 1:** Comparison of Biochemical parameters in Cases and Controls

Patients	Cases (N=150)	Controls (N=150)	*t* or χ^2^	*P* value
**Age (years)**	52.68±5.440	52.00±5.188	1.108	0.269
**Gender**				
Male (n,%)	116 (77.3%)	70 (46.7%)	29.938	**<0.0001**
Female (n,%)	34 (22.7%)	80 (53.3%)		
**BMI (Kg/m2)**	24.80±4.595	23.71±1.233	2.797	**0.005**
**HDL (mg/dL)**	42.98±3.488	56.11±9.848	15.388	**<0.0001**
**LDL (mg/dL)**	150.35±30.270	71.43±28.916	23.09	0
**Total cholesterol (mg/dL)**	216.11±30.922	153.59±22.173	20.126	0
**Triglyceride (mg/dL)**	113.89±14.683	129.48±62.532	2.972	**0.003**
**VLDL (mg/dL)**	22.80±3.061	25.92±12.449	2.981	**0.003**

### Genotyping

Genotypes & alleles frequencies of KCNJ11 and eNOS genes in cases and healthy controls

The results were analyzed as allelic and genotypic frequencies of the KCNJ11E23K and eNOSG894T polymorphisms (Table 2). The frequency of KK genotype of KCNJ11E23K polymorphism was found to be highly significant in cases with 25 fold higher risk of CAD (p≤0.0001) in comparison to control. Similarly the frequency of K* genotype of KCNJ11E23K polymorphism was also found to be highly significant in cases with 3.45 fold higher risk of CAD (p≤0.0001). EK in combination with KK (EK+KK) was also found highly significant in cases with 6.30 fold higher risk of CAD. However the frequency of EK genotype of KCNJ11E23K polymorphism was moderately significant in control in comparison to CAD cases (p=0.001). No significant association was found in EE polymorphisms between cases and control. In case of eNOSG894T polymorphisms no significant association was found between cases and control.

**Table 2:** Genotypes & alleles frequencies of *KCNJ11* and *eNOS* genes in cases and healthy controls

Genotypes	Cases		Controls		OR (95%CI)	*P* value
	Number (N=150)	Frequency (%)	Number (N=150)	Frequency (%)		
***eNOS* G894T**						
GG	95	63.3	101	67.3	1(Ref)	
GT	48	32	42	28	1.215 (0.737–2.003)	0.445
TT	7	4.7	7	4.7	1.063 (0.359–3.145)	0.912
GT+TT	55	36.7	49	32.7	1.19 (0.741–1.921)	0.467
Allele						
G*	238	79.3	244	81.33	1(Ref)	
T*	62	20.7	56	18.67	1.14 (0.759–1.698)	0.538
***KCNJ11 E23K***						
EE	9	6	43	28.7	1(Ref)	
EK	73	48.7	94	62.7	**3.710 (1.700–8.101)**	**0.001**
KK	68	45.3	13	8.7	**24.991 (9.843–63.452)**	**<0.0001**
EK+KK	141	94	107	71.3	**6.30 (2.941–13.478)**	**<0.0001**
Allele						
E*	91	30.33	180	60	1(Ref)	
K*	209	69.67	120	40	**3.45 (2.458–4.828)**	**<0.0001**

### Association of genotype eNOS and KCNJ11 in male and female cases & controls

The results were analyzed as association of genotype KCNJ11E23K and eNOSG894T in male and female cases (Table 3). In case of males the frequency of EK genotype of KCNJ11E23K polymorphism was found to be moderately significant in cases with 0.23 fold higher risk of CAD (p=0.004) in comparison to control. The frequency of KK genotype was found to be highly significant in cases with 0.02 fold higher risk of CAD (p≤0.0001) Similarly, the frequency of K* genotype of KCNJ11E23K polymorphism was also found to be highly significant in cases with 0.25 fold higher risk of CAD (p≤0.0001). Whereas no significant association was found in EE and E* genotype between cases and control. In case of females, the frequency of KK genotype of KCNJ11E23K polymorphism was found to be moderately significant in cases with 0.09 fold higher risk of CAD (p=0.001) in comparison to control. Similarly the frequency of K* genotype of KCNJ11E23K polymorphism was also found to be moderately significant in cases with 0.35 fold higher risk of CAD (p=0.001). However no significant association was found in EE, EK and E* genotype betweecases and control. Again in case of eNOSG894T polymorphisms no significant association was found between cases and control in both males and females.

**Table 3:** : Association of genotype *eNOS* and *KCNJ11* in male and female cases & controls

Genotypes	Male		OR (95%CI)	*P* value	Female		OR (95%CI)	*P* value
	Cases (n=116)	Controls (70)			Cases (n=34)	Controls (n=80)		
***eNOS* G894T**								
GG	77 (66.4)	48 (68.6)	1(Ref)		18 (52.9)	53 (66.2)	1(Ref)	
GT	34 (29.3)	18 (25.7)	0.85 (0.432–1.669)	0.635	14 (41.2)	24 (30.0)	0.58 (0.249–1.360)	0.212
TT	5 (4.3)	4 (5.7)	1.28 (0.328–5.017)	0.72	2 (5.9)	3 (3.8)	0.51 (0.079–3.297)	0.479
Allele								
G*	188 (81.0)	114 (81.4)	1(Ref)		50 (73.5)	130 (81.2)	1(Ref)	
T*	44 (19.0)	26 (18.6)	0.97 (0.569–1.668)	0.925	18 (26.5)	30 (18.8)	0.64 (0.328–1.252)	0.193
***KCNJ11 E23K***								
EE	6 (5.2)	21 (30.0)	1(Ref)		3 (8.8)	22 (27.5)	1(Ref)	
EK	57 (49.1)	46 (65.7)	**0.23 (0.086–0.619)**	**0.004**	16 (47.1)	48 (60.0)	0.41 (0.108–1.550)	0.189
KK	53 (45.7)	3 (4.3)	**0.02 (0.004–0.071)**	**<0.0001**	15 (44.1)	10 (12.5)	**0.09 (0.021–0.387)**	**0.001**
Allele								
E*	69 (29.7)	88 (62.9)	1(Ref)		22 (32.4)	92 (57.5)	1(Ref)	
K*	163 (70.3)	52 (37.1)	**0.25 (0.161–0.390)**	**<0.0001**	46 (67.6)	68 (42.5)	**0.35 (0.195–0.642)**	**0.001**

## Discussion

CAD has been found to be the major cause of mortality and morbidity in human. Widespread studies perused on the epidemiology of CAD have reported the incidence of CAD in almost all parts of the world. In the developing countries, these cardiovascular diseases (CVD) are categorized as epidemic. Several case-control studies, consortia, genome-wide association analysis and epidemiological studies have been conducted to understand the different aspects of this disease. Conventional threats include type 2 diabetes (T2D), arterial hypertension, dyslipidemia and cigarette smoking^27^.

Single nucleotide polymorphisms (SNPs) could serve as prognostic factor as they influence disease incidence and can be employed in everyday clinical practice and decision making; hence, it is essential to identify those SNPs that have strongest predisposition. Hence, this article reveals the allelic and genotypic frequencies of the KCNJ11E23K and eNOSG894T polymorphisms as they are related to atherosclerotic consequences which lead to CAD.

A point mutation found at nucleotide 894 in exon 7 where guanine (G) shifts to thymine (T) in the eNOS gene transforms the coding sequence from glutamic acid to aspartic acid in codon 298 (Glu298Asp, also known as the G894T, rs1799983)28. Recently through several reports it was concluded that eNOSG894T (Glu-298→Asp) mutation at exon 7 of the eNOS gene is related to coronary spasm^28^, myocardial infarction (MI)^29^, ^30^, and hypertension^31^. Previous reports, illustrate that the Glu2983Asp polymorphism is significantly associated with the presence of CAD^32^. However our results did not show any significant association in eNOS genotype between cases and control. Our results were also not discordant with the outcomes of Chang et al and Kerkeni et al^33^,^34^ who found a significant correlation between Glu298Asp polymorphism and CAD. Although Abolhalaj et al also revealed that the difference in eNOS gene expression was not statistically significant between patients and control^35^.

Another gene that has been focused in this article is KCNJ11 gene as it also guides the formation of ATP-sensitive potassium (K-ATP) channel in cardiomyocytes. These ATP sensitive channels play important role in CAD^20^. Our result stated that the frequency of KK genotype and K* genotype of KCNJ11E23K polymorphism was found to be highly significant in cases with 25 and 3.45 fold higher risk respectively in CAD (p≤0.0001) in comparison to control. EK in combination with KK (EK+KK) was also found highly significant in cases with 6.30 fold higher risk of CAD. However the frequency of EK genotype of KCNJ11E23K polymorphism was moderately significant in control in comparison to CAD cases (p=0.001). No significant association was found in EE polymorphisms between cases and control. In case of males the frequency of EK genotype of KCNJ11E23K polymorphism was found to be moderately significant in cases with 0.23 fold higher risk of CAD (p=0.004) in comparison to control and the frequency of KK genotype was found to be highly significant in cases with 0.02 fold higher risk of CAD (p≤0.0001) Similarly, the frequency of K* genotype of KCNJ11E23K polymorphism was also found to be highly significant in cases with 0.25 fold higher risk of CAD (p≤0.0001). Whereas no significant association was found in EE and E* genotype between cases and control. In case of females, the frequency of KK genotype of KCNJ11E23K polymorphism was found to be moderately significant in cases with 0.09 fold higher risk of CAD (p=0.001) in comparison to control. Similarly the frequency of K* genotype of KCNJ11E23K polymorphism was also found to be moderately significant in cases with 0.35 fold higher risk of CAD (p=0.001). However no significant association was found in EE, EK and E* genotype between cases and control. Fedele et al aimed to evaluate the clinical aspects of SNPs in genes associated with ischemic heart disease in which KCNJ11 was one of the candidate genes. Through this study he observed a significant difference (p<0.05) for SNP rs 5219 of KCNJ11 gene. However, he also deduced that SNP 5219 of KCNJ11 has inverse correlation with cardiovascular dysfunction as its frequency was higher in control group^36^. Their results suggest that genetic polymorphism could also be used to identify patients with low risk of CAD regardless of presence T2DM and dyslipidemia. Whereas, our results showed correspondence with the results of Xiong et al who demonstrated that the E23K gene polymorphism in KCNJ11 gene is related to susceptibility to CAD^37^. Although, on the other hand, Samadikuchaksaraei et al as did not find any association between CAD and E23K polymorphism in Iranian patients^38^. The sample size of this polymorphism was small; therefore we cannot rule out the possibility of the results being affected. Hence, genome-wide association study on distinct population with large sample size is suggested as a more widespread approach answering many more questions.

## Conclusion

Polymorphism study can serve as prognostic factor as it influences disease occurrence and could aid in everyday clinical practice hence, it is essential to identify those polymorphisms that have strongest predisposition. Further studies on these genes in specific and large populations are required to improve our understanding regarding the role of gene polymorphisms and also to achieve any possible clinical significance in terms of prognosis or therapeutic intervention for CAD patients.

## Figures and Tables

**Fig: 1:**
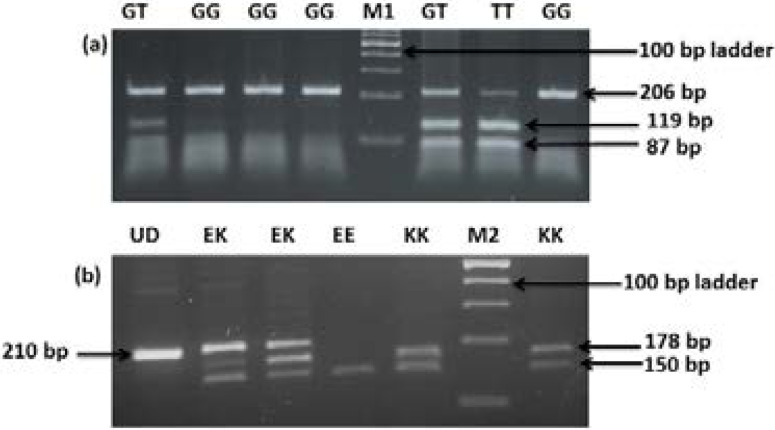
**Restriction fragment length polymorphism analysis for determination of genotype. (a) For *eNOS*** gene polymorphisms, The GT genotype shows two bands of 206 bp and 119 bp; The GG genotype shows single band of 206 bp, The TT genotype shows two bands of 119 bp and 87bp. (b) For *KCNJ11* gene polymorphisms, UD shows Undigested band of 210bp, the EK genotype shows three bands of 210 bp, 178 bp and 150 bp; the EE genotype shows one band of 150 bp; The KK genotype shows two bands of 178 bp and 150 bp. M1 AND M2 are 100 bp molecular ladder.
